# A Nationwide Cross-Sectional Online Survey on the Treatment of COVID-19-ARDS: High Variance in Standard of Care in German ICUs

**DOI:** 10.3390/jcm10153363

**Published:** 2021-07-29

**Authors:** Steffen Dickel, Clemens Grimm, Maria Popp, Claudia Struwe, Alexandra Sachkova, Martin Golinski, Christian Seeber, Falk Fichtner, Daniel Heise, Peter Kranke, Winfried Meissner, Sven Laudi, Sebastian Voigt-Radloff, Joerg Meerpohl, Onnen Moerer, on behalf of the German CEOsys Study Group

**Affiliations:** 1 Department of Anesthesiology and Intensive Care Medicine, University Medical Center Göttingen, 37085 Göttingen, Germany; steffen.dickel@med.uni-goettingen.de (S.D.); clemens.grimm@med.uni-goettingen.de (C.G.); claudia.struwe@med.uni-goettingen.de (C.S.); alexandra.sachkova@med.uni-goettingen.de (A.S.); martin.golinski@med.uni-goettingen.de (M.G.); dheise1@gwdg.de (D.H.); 2 Department of Anaesthesiology, Intensive Care, Emergency and Pain Medicine, University Hospital Wuerzburg, 97080 Wuerzburg, Germany; popp_m4@ukw.de (M.P.); kranke_p@ukw.de (P.K.); 3 Department of Anesthesiology and Intensive Care Medicine, University Medical Center Leipzig, 04103 Leipzig, Germany; christian.seeber@medizin.uni-leipzig.de (C.S.); falk.fichtner@medizin.uni-leipzig.de (F.F.); sven.laudi@medizin-uni-leipzig.de (S.L.); 4 Department of Anesthesiology and Intensive Care Medicine, University Medical Center Jena, 07743 Jena, Germany; winfried.meissner@med.uni-jena.de; 5 Institute for Evidence in Medicine, Faculty of Medicine and Medical Center, University of Freiburg, 79106 Freiburg, Germany; voigt-radloff@ifem.uni-freiburg.de (S.V.-R.); meerpohl@ifem.uni-freiburg.de (J.M.); 6 Cochrane Germany, Cochrane Germany Foundation, 79110 Freiburg, Germany

**Keywords:** COVID-19, ICU, ventilation, PEEP, tracheotomy, variance of care

## Abstract

Introduction: Coronavirus disease (COVID-19) has recently dominated scientific literature. Incomplete understanding and a lack of data concerning the pathophysiology, epidemiology, and optimal treatment of the disease has resulted in conflicting recommendations. Adherence to existing guidelines and actual treatment strategies have thus far not been studied systematically. We hypothesized that capturing the variance in care would lead to the discovery of aspects that need further research and—in case of proven benefits of interventions not being performed—better communication to care providers. Methods: This article is based on a quantitative and qualitative cross-sectional mixed-methods online survey among intensive-care physicians in Germany during the COVID-19 pandemic by the CEOsys (COVID-19 Evidence Ecosystem) network, endorsed by the German Interdisciplinary Association for Intensive Care and Emergency Medicine (DIVI) conducted from December 3 to 31 December 2020. Results: We identified several areas of care with an especially high variance in treatment among hospitals in Germany. Crucially, 51.5% of the participating ICUs (*n* = 205) reported using intubation as a last resort for respiratory failure in COVID-19 patients, while 21.8% used intubation early after admission. Furthermore, 11.5% considered extracorporeal membrane oxygenation (ECMO) in awake patients. Finally, 72.3% of respondents used the ARDS-network-table to titrate positive end-expiratory-pressure (PEEP) levels, with 36.9% choosing the low-PEEP table and 41.8% the high-PEEP table. Conclusions: We found that significant differences exist between reported treatment strategies and that adherence to published guidelines is variable. We describe necessary steps for future research based on our results highlighting significant clinical variability in care.

## 1. Introduction

Since the severe acute respiratory syndrome coronavirus *2* (SARS-CoV-2) outbreak in Wuhan, China in late 2019, the virus has spread around the world and has now infected over 3.5 million people in Germany and approximately 160 million people worldwide as of 14 May 2021 [1,2]. Infection with the virus can lead to Coronavirus Disease 2019 (COVID-19), which can present with manifestations ranging from mild cold symptoms to severe sepsis, acute respiratory distress syndrome (ARDS), and multi-organ failure [3,4,5,6]. As of the submission of this article, more than 3.33 million people have died from this disease [2]. According to the U.S. Centers for Disease Control and Prevention, approximately 4.9 to 11.5% of COVID-19 patients need to be admitted to the intensive care unit (ICU) [7]. Here, advanced treatment includes measures such as high-flow nasal cannula (HFNC), invasive ventilation, proning, and extracorporeal procedures [3,8]. Meanwhile, numerous recommendations describing the treatment of COVID-19 patients have been published [9,10,11,12]. The lack of robust evidence is reflected by rapidly changing recommendations in key areas of care, such as pharmacotherapy and ventilatory management [13,14,15,16,17]. A distinction should be made between variance in recommendations and variance in guideline adherence. Differences in adherence to recommendations also speak to a lack of trustworthiness of evidence-based recommendations.

A network of 20 university medical centers and other partners has been formed in Germany (COVID-19 Evidence Ecosystem network, CEOsys), whose goals are to (1) synthesize the current state of research; (2) to support development of evidence-based recommendations to improve management of the pandemic in Germany; and (3) to detect research gaps to facilitate evidence-based research.

The critical care experts of the CEOsys network hypothesized that there is a high uncertainty regarding the optimal treatment strategy regarding critically ill COVID-19 patients. Our cross-sectional survey aimed to assess the current national standards of care for critically ill COVID-19 patients and to identify variance in core clinical treatment strategies. We aimed to identify gaps of evidence to promote further clinical research. The results of the survey concerning questions other than respiratory management will be published elsewhere.

## 2. Methods

This study is a qualitative and quantitative analysis of an anonymous cross-sectional mixed-methods survey conducted online from 3 to 31 December 2020 by the CEOsys network, endorsed by the German Interdisciplinary Association for Intensive Care and Emergency Medicine (DIVI). Within CEOsys scientifically standardized PICO-style questions (PICO; P—patient, population of problem, I—intervention, C—comparison/control, O—outcome or endpoint) were created. These can also be applied to further analysis, e.g., in the setup of systematic reviews.

### 2.1. Survey Format

This was a closed-access survey consisting of 9 introductory questions, 16–21 (adaptive) questions on ventilation, 4–7 (adaptive) questions on medication, and 7 questions on protective and isolation measures on 10 pages (3–5 questions per page).

The questions were designed by a total of 12 intensivists in several online conferences. The content refers to the relevant topics for the experts at that time. The individual questions were discussed and were included in the questionnaire after agreement.

The questions were designed as multiple-choice and multiple-select questions. The items’ order of appearance was not randomized. Completeness checks before submitting were carried out and selection of at least one response option was enforced [18]. SoSci-Survey is a professional online survey tool that was used as a secure online platform to create and distribute the survey questions.

### 2.2. Pre-Survey Assessment and Data Analysis

The survey was targeted to be completed within a maximum of 10 min. Participants were informed about the approximated time duration, data management, data storage, the investigators, and the purpose of the study according to the CHERRIES criteria for online surveys [19]. Prior to distribution of the survey, the time required for completion was piloted by 9 specialists, yielding an effort of approximately 9 min and 47 s. Based on this finding, the questionnaire was limited to 44 questions.

The objective was to assess the current standards in intensive care units for providing care for critically ill patients suffering from COVID-19:

1. Usage of HFNC
2. Mechanical ventilation
3. Prone positioning
4. Tracheotomy

The survey was aimed at the leading physicians of each German ICU. Lead status and experience in treating COVID-19 patients were queried. A code consisting of parts of the postal code, telephone number, floor, and department was used to exclude duplicate participation. Neither cookies, IP check, or log files were used. Incomplete surveys were included into the analysis. Duplicate data records, and those non-related to the treatment of COVID-19 patients, were excluded.

### 2.3. Questionnaire

The questionnaire used can be found in Table 1.

### 2.4. Recruitment

The DIVI register comprises of 1340 sites reporting their capacity for intensive care beds on a daily basis. These 1340 ICUs included units that did not treat COVID-19 patients or admitted only specialty-specific patients (for example, pediatric ICU). The invitation for our online survey was sent to leading ICU physicians via the DIVI’s email distribution list (for invitational email please see Appendix A). Our survey was not advertised publicly. The invitation to the survey was sent together with that to another study.

## 3. Results

**Figure 1:**
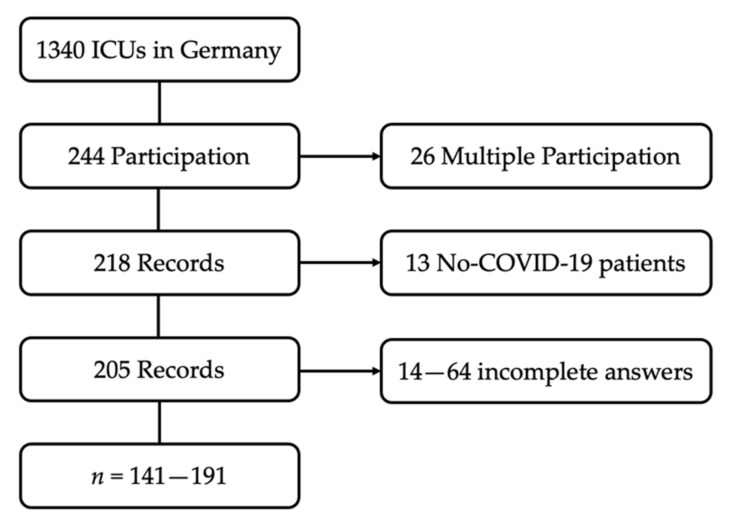
Flow Chart: composition of the study population.

Of the 1340 German registered ICUs, 244 (18%) participated in the online survey. The questionnaire was fully completed 141 times (see also Figure 1). At the beginning of the study period, 32,481 COVID-19 ICU treatments had been completed in Germany [20]. Based on our data, the participating ICUs had treated at least 6659 of these patients. Accordingly, the survey covers about 20.5% of the patients who had been treated up to that time. It can be assumed that this proportion is even higher. Some of the ICUs participating in the study did not provide us with the number of patients treated; the number of total patients in Germany includes double counting, mainly due to interhospital transfers.

Completion of the entire survey took an average of 9 min and 7 s ± 6 min and 10 s (mean value (MV) ± standard deviation (SD)). The completion rate of the survey was 66.3%. On average, 36.3 ± 33.7 COVID-19 (MV ± SD) patients had been treated in the participating ICUs by the time the survey was completed. It was discovered that 57.6% of respondents had to estimate the number. Over 90% of ICUs had permanent access to continuous renal replacement and advanced hemodynamic monitoring. Furthermore, 44% had access to extracorporeal membrane oxygenation (ECMO), while 10.5% had pumpless extracorporeal lung assist (pECLA) at their disposal. Detailed results can be found in Table 2.

### 3.1. Main Findings

**Figure 2:**
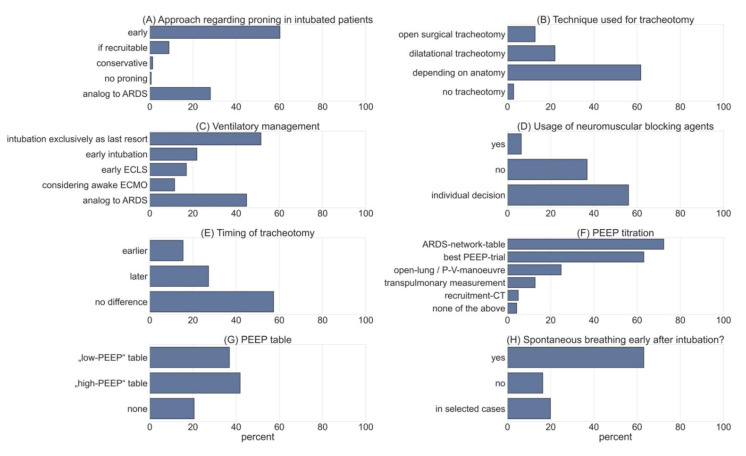
Shows the results obtained from selected questions of the survey. (**A**) Data obtained on intubated patients with regards to proning (multiple-choice, *n* = 146). (**B**) Technique used for tracheotomy (multiple-choice, *n* = 141). (**C**) Approach to respiratory management in COVID-19 patients (multi select; *n* = 165). (**D**) Usage of neuromuscular blockade (NMB) > 24 h (multiple-choice, *n* = 141). (**E**) Timing of tracheotomy compared to ARDS patients (multiple-choice, *n* = 136). (**F**) Approach to PEEP titration (multiple-select, *n* = 141). (**G**) Usage of PEEP table from ARDS-network (multiple-choice, n = 141). (**H**) Data on spontaneous breathing early after intubation (multiple-choice, *n* = 141).

A total of 165 participants answered questions regarding management of acute respiratory failure. Notably, 51.5% stated that intubation was performed as a last resort in patients with progressing respiratory failure under HFNC or non-invasive positive pressure ventilation (NIPPV). This contrasts with 21.8% of participants who explicitly considered intubation and invasive ventilation as a preventive measure. Notably, only 44.8% claimed to guide treatment, according to the level 3 Guideline on ARDS [21]. Furthermore, 28 participants (17%) considered early implementation of ECMO/pECLA in ventilated patients, while 11.5% performed ECMO/pECLA in patients that had not been intubated and were breathing spontaneously (see Figure 2).

Most of the ICU respondents (90.3%, to be exact) used HFNC to assist gas exchange. In addition, 27.3% used alternative interfaces, such as a helmet for NIPPV ventilation, and 87.9% indicated disturbance of consciousness as the main discontinuation criterion for NIPPV. The blood pH was mentioned several times as an important parameter as well, but was not queried specifically. Cut-off values for discontinuation of NIPPV therapy were reported to be 115.40 ± 40.56 mmHg (MV ± SD) for Horovitz, 32.02 ± 8.13/min (MV ± SD) for respiratory rate, and 63.67 ± 18.01 mmHg (MV ± SD) for pCO_2_. Notably, the free text comments repeatedly stated that there was no single parameter guiding treatment alone.

### 3.2. PEEP

We assessed the tools intensivists used to guide their treatment regarding PEEP using a multiple-select approach. A total of 72.3% stated that they used the ARDS-network chart to determine optimal PEEP levels, and 63.1% used “best-PEEP-trials” guiding their treatment. Open-lung tools were used by 24.8% of respondents, while 12.8% used advanced techniques such as measurement of transpulmonary pressures via esophageal feeding tubes. Only 5.0% performed recruitment computer tomography (CT) scans. When using the ARDS-network PEEP charts, 36.9% used the “low-PEEP” approach, while 41.8% preferred the “high-PEEP” chart.

### 3.3. Neuromuscular Blockade

Only 6.4% used continuous neuromuscular blockade in intubated patients in acute respiratory failure over a period of more than 24 h. Moreover, 56% only used this strategy in individual cases, leaving 36.9% of respondents who did not use neuromuscular blockade in an attempt to facilitate oxygenation. In addition, 63.1% aimed to enable assisted spontaneous breathing in the first 24 h after intubation; 16.3% did not follow this strategy, while 19.9% decided on a case-to-case basis (see Figure 2).

A total of 43.2% of respondents claimed to instruct awake patients to self-prone during their stay in the intensive-care unit, while 38.0% used 90–130° proning. Additionally, 19.2% did not use proning in patients that were not intubated. As soon as intubation had been deemed necessary, 60.3% used proning as a preventive measure even before the P/F ratio dropped below 150. Proning was used in 8.9% of patients who had documented potential for recruitment (see Figure 2). Furthermore, 28.1% responded that they did not differ from current strategies for proning, as specified in the current German guideline on invasive ventilation and ECMO in acute pulmonary failure [21].

### 3.4. Tracheotomy

We obtained answers on timing of tracheotomy and the techniques that were used preferentially. Interestingly, 12.8% of the respondents stated that they preferred to use surgical techniques to reduce production of aerosols. However, 22.0% stated that they used dilatational techniques for the same reason. The majority (61.7%) made the decision based upon patients’ characteristics, such as anatomy of the neck. Only 2.8% did not perform any tracheotomies in COVID-19 patients (see Figure 2).

By comparing COVID-19 patients to patients with ARDS from other causes, we found that 57.4% of respondents did not change their timing of tracheotomy. In addition, 15.4% stated they would perform the procedure earlier, in contrast to 27.2% that claimed to indicate tracheostomy later in the course of the disease compared to other patients with ARDS.

## 4. Discussion

We surveyed more than 200 intensive care units in Germany on current practices in patient care. High variability in several clinically relevant areas was found.

The COVID-19 pandemic continues to confront healthcare systems globally with unprecedented challenges [22]. Due to the high number of cases of COVID-19 clustered in time, some regions of the world experienced overcrowding of hospitals [23]. In these regions, triage and high mortalities from the SARS-CoV-2 virus occurred [23]. In addition, the considerable psychological burden of the pandemic situation for the population, especially for health care workers (HCWs) working at the limit, should be mentioned [23,24,25].

The need for guidance in the treatment of patients admitted to the intensive care unit remains high. We were able to show that, regarding key components of care, significant differences exist in the treatment of COVID-19 patients on German ICUs. Based on our findings, there are several areas where clinical consensus is currently lacking. This includes, but is not limited to, the optimal timing of intubation (51.5% stated to intubate only as a last resort measure, while 21.8% of respondents intubated early in the disease progress), conversion to spontaneous breathing (only 63.1% stated to pursue spontaneous breathing in intubated patients), and PEEP titration. Of course, the optimal usage of PEEP is the subject of a debate that has been ongoing for decades, irrespective of COVID-19 [26,27,28,29]. About 20.6% preferred an individualized approach to PEEP settings; 36.9% used the low PEEP table compared to 41.8% using the high PEEP table. This reveals that the amount of confusion is equally high regarding COVID-19-related respiratory failure as in patients suffering from ARDS of other causes. It is important to keep in mind that the resulting variance in applied PEEP might be especially high in patients with a less severe hypoxemic respiratory failure. Taking into account the recently described changes in pathophysiology [30] that result in different PEEP requirements, the effects might well be deleterious in the clinical setting. There is an urgent need to create evidence for a PEEP concept that applies to the different stages of COVID-19-related acute respiratory failure.

Despite the initial fear for aerosol generation by means of HFNC, we found that more than 90% used this technique in COVID-19 patients. This reflects current evidence that does not implicate increased aerosol exposure by HFNC compared to NIPPV even in high flow rates [31].

We found that 51.5% of participating ICUs tried to extend time spent on HFNC to avoid invasive ventilation. As mentioned before, the threshold for invasive ventilation remains unclear due to the lack of trustworthy evidence. This is reflected by different recommendations in the current guidelines which are often derived from nonCOVID ARDS guidelines and are extrapolated to the treatment of COVID-19 patients [9,10,11,12]. It is unclear whether intubation was postponed during the time of our study due to resource reasons. Although staffing levels were reported to be inadequate in some cases, this does not seem very plausible, as ICU capacities were strained but did not reach their limits during any of the COVID waves in Germany [32,33]. It is conceivable that international media reports have led to an overly conservative approach to airway management in COVID-19 patients in Germany in order to conserve resources. Several guidelines recommend against the routine use of continuous infusions of neuromuscular blocking agents (NMBAs) in mechanically ventilated patients with COVID-19 [10]. In this study, only 6.4% of the participants use NMBA to improve ventilation, thus reflecting the opinion in recent literature [10,34]. However, 56% of ICUs used NMBA in selected cases. This fits with the German Level 3 guideline that allows administration as an option in complex cases [35]. We know by now that NMBAs are well tolerated for a short period of time and can be used to prevent high respiratory rates, high tidal volumes, and high inspiratory efforts that lead to irreparable lung injury [36]. Although the administration of NMBA is promising and should be considered, especially in the early phase of ARDS, the evidence is weak and it is recommended to optimize ventilation and sedation first [36]. Evidence suggests that NMBA administration should then be planned in such a way that early spontaneous breathing can be established [37]. In this context, we found that 63.1% aimed to enable spontaneous breathing early after intubation. This implies that more than a third of respondents did not specifically aim to adjust the sedation and analgesic regimen sufficiently to facilitate spontaneous breathing.

Prone positioning is considered a low-risk and low-cost intervention to improve oxygenation in ARDS patients [38]. Notably, evidence supporting this notion is small. However clinical experts recommend the usage of prone positioning in COVID-19 patients, even in patients that are not mechanically ventilated. Respondents used proning in 81.2% of cases of non-intubated patients with relevant respiratory impairment. In intubated patients, 60.3% of respondents used prone positioning early in the disease, while only 2.1% hesitated or never used proning at all.

Although large studies that confirm the benefit of proning in COVID-19 patients are still lacking at this point, clinical practice shows that proning improves the P/F ratio and, thus, can reduce the lung stress after optimizing ventilator pressures [39,40]. Positive hemodynamic effects and less cardiac arrests should also not be ignored [41,42]. Evidence for the positive effect of proning in awake patients with COVID-19 already exists [43]. The high proportion of ICUs performing proning does not seem to be surprising considering the negligible risk and cost of this intervention, which concurs with specific recommendations in the current literature [9,10,11]. Further robust and large-scale studies indicating clear benefit for patients are needed.

Conflicting guidance exists concerning the technique and timing for tracheotomy in COVID-19 patients [44,45]. Several arguments against early tracheotomy have been proposed, e.g., the idea of a lower viral load in later stages of the disease to reduce occupational risks in healthcare workers [46,47]. However, recommendations for an early intervention have been put forward on the grounds of facilitating weaning, avoiding respiratory muscle atrophy, and freeing up scarce intensive care resources during the pandemic [46,47]. Similar arguments have been made in the context of technique that should be used. This unsolved debate is reflected by our data. Concerning the timing of tracheotomy, it appears that 42.6% of respondents deliberately differ from standard practice in ARDS patients, performing tracheotomy either earlier or later. Similar disparity was seen in the description of techniques used (e.g., surgical or dilatational).

Several countries have published guidelines to provide evidence-based treatment and inform caregivers and patients alike. Due to the staggering amount of new clinical research issued, daily treatment strategies need to be updated frequently and clinicians need to be forced to keep up to date with the current evidence. It is thus unsurprising that our survey reveals a significant variance in the treatment concepts for critically ill COVID-19 patients in Germany.

Considering the fact that national guidelines regarding ARDS have been in place for several years [21] and that the national level 3 guideline for COVID-19 has already been recently published [9], these differences within the German healthcare system are quite remarkable. There are several explanations: it is known that research-to-bedside time can be as high as 17 years [48]. Implementation of recent research findings can be difficult when there are established treatment protocols.

During the ongoing public health emergency, high strain is being felt upon the healthcare system, not only due to the demanding care for critically ill individuals but also because of psychological burden and economical challenges [49,50]. It is thus possible that our results may simply be due to an ongoing surge of critically ill patients and a clinical workforce suffering from insufficient funding, staffing, and time [25,51,52]. However, it needs to be questioned if the high variance is acceptable in the context of an ongoing medical and epidemiological emergency and whether implementation of standards could be faster.

Further efforts are needed to disseminate established recommendations effectively and to promote a standardized care approach while leaving room for individual considerations.

## 5. Limitations

The current study included a limited sample size and the naturally rigid structure of an online survey. This includes a moderate response rate that led to a semi-representative sample size. Additionally, it should be noted that small hospitals (<200 beds), in particular, are underrepresented in the study, which may additionally influence responses. The exact number of beds in the individual intensive care units was also not queried. It must be assumed that, due to the nature of our survey, volunteer bias was introduced into the sample. It should be considered that ICUs with the highest workloads were possibly unable to respond to our survey due to a lack of time and resources.

Having sent the questionnaire to the leading physicians only, answers might be biased towards ideal or at least the official standards of the given ICU and actual implementation of recommended therapies may be worse than, or at least differ from, our findings. Since practice and perception might differ considerably, the variance in bedside care is expected to be higher than reported [53].

Several respondents have mentioned the ‘rigid’ frame of a multiple-choice question to be a hindrance in describing their actual management of COVID-19 patients. 

The limited responses of the survey are also biased, which leads to a limited representation of the reality in German ICUs. Considering the large number of patients treated by the responding ICUs from all over Germany, we can still confidently state to have captured a realistic description of the actual concepts of care delivered to COVID-19 patients in Germany.

## 6. Conclusions

There are large differences in the treatment and management of acute respiratory failure in critically ill COVID-19 patients in German ICUs. Reasons for these variances in care might include the vast amount of published material and recommendations, the delay in adaptation of new concepts, but also the persistent lack of clear evidence and numerous research gaps in the critical care setting. To improve outcomes in COVID-19 patients, national and joint international efforts are required to generate evidence for the ideal therapeutic concepts regarding respiratory treatment. Moreover, focus should be laid upon supporting the fast implementation of these concepts at the bedside.

## Figures and Tables

**Table 1:** Questions asked during the online survey. (*) for questions with multiple answers possible.

Questions			
Have you previously treated COVID-19 patients in your ICU?			
Yes	No		
Please tell us how many COVID-19 patients you have provided on your ICU to date.			
Exact number	Estimated number	Specification not possible	
Please list the number of beds in your hospital.			
<200	200–600	600–1000	>1000
Please list any special technical equipment available in your ICU. (*)			
Extracorporeal membrane oxygenation (ECMO)		Pumpless extracorporeal membrane oxygenation (pECLA)	
Renal replacement therapy (24 h available)		Advanced hemodynamic monitoring (PiCCO, Swan–Ganz catheter)	
Advanced respiratory monitoring (NAVA, EIT, etc.)		Adaptive ventilation modes (NAVA, PAV, PAV+, etc.)	
NO inhalation therapy		Cytokine elimination procedures	
Please describe your approach to ventilation in COVID-19 patients compared to other patients with respiratory failure. (*)			
Intubation exclusively as last resort (prolonged NIPPVV, HFNC etc.)		Early decision for intubation and invasive ventilation	
Early decision for extracorporeal procedures (ECMO, pECLA)		Performance and consideration of “awake ECMO”.	
Basically, no difference to the procedure described in the German level 3 guideline for ARDS patients.			
Describe the discontinuation criteria for NIV ventilation in COVID-19 patients.			
Consciousness disorder	Respiratory rate	Clinical assessment of the respiratory work	
Rapid-Shallow-Breathing-Index	CO_2_ elimination disorder	Horovitz/oxygenation index	Work of breathing
If you are using RSBI as a discontinuation criterion for NIV therapy, explain your threshold.			
If you are using Horovitz as a discontinuation criterion for NIV therapy, explain your threshold.			
If you are using respiratory rate as a discontinuation criterion for NIV therapy, explain your threshold.			
If you are using work of breathing as a discontinuation criterion for NIV therapy, explain your threshold.			
If you are using pCO_2_ as a discontinuation criterion for NIV therapy, explain your threshold.			
What alternative procedures are used instead of invasive ventilation in your ICU for critically ill COVID-19 patients. (*)			
Oxygen therapy only		High-flow nasal oxygen (HFNC)	
Conventional non-invasive ventilation via mask		Alternative NIV interface (helmets, etc.)	
If you use an HFNC, what flow rates are used in critically ill COVID-19 patients?			
HFNC as usual		No HFNC due to potential aerosol exposure for personnel	
Reduced flow rates compared to non-COVID to reduce aerosol production			
Please describe your approach to proning in non-intubated COVID-19 patients with severely impaired lung function in your ICU.			
Instruction for self-positioning of patients in prone position (“self-proning”)			
130°-positioning or lateral-positioning		No proning in patients without invasive ventilation	
Please describe your approach to proning in intubated COVID-19 patients with severely impaired lung function in your ICU.			
Early proning (already above P/F ratio of 150)		Prone positioning only in patients with proven potential of recruitment	
Restrained indication for proning		No proning	
No difference to the described procedure in the German level 3 guideline for ARDS patients.			
What tools do you use to adjust PEEP in COVID-19 patients? (*)			
ARDS Network Table	Best PEEP-Trial	Open-lung-tool/P-V maneuver	Recruitment CT-Scan
None of these methods	Transpulmonary pressure measurement		
If you are using the ARDS network table to set PEEP, which table are you using as?			
low PEEP table	high PEEP table	No use of the PEEP table	
Are you using permanent (>24 h) neuromuscular blockade in COVID-19 patients to improve ventilation?			
Yes	No	Only in individual cases	
In COVID-19 patients * with severe ARDS, are you already early aiming for spontaneous breathing?			
Yes	No	Only in individual cases	
Which tracheostomy procedure do you use for critically ill COVID-19 patients?			
Preferred surgical tracheostomy to reduce aerosol exposure to staff		Preferred puncture tracheotomy to reduce aerosol exposure to staff	
Both procedures, choice based on anatomic structures		No tracheotomy in COVID-19 patients	
Please describe the tracheostomy timing in COVID-19 patients compared to other ARDS patients.			
Earlier	Later	No difference	

**Table 2:** Results of the online survey. Data in absolute number or mean value ± standard deviation. The original questions were asked in German. English translations are shown here. (*) for questions with multiple answers possible.

Question	*n*	*n* (%) or MV ± SD
Have you previously treated COVID-19 patients in your ICU?	218	
Yes		205 (94.0)
No		13 (6.0)
Please tell us how many COVID-19 patients you have provided on your ICU to date.	191	
Exact number	75	30.84 ± 30.16
Estimated number	110	40.25 ± 35.38
Specification not possible	4	
Please list the number of beds in your hospital.	191	
<200		25 (13.1)
200–600		69 (36.1)
600–1000		32 (16.8)
>1000		61 (31.9)
Please list any special technical equipment available in your ICU. (*)	191	
Extracorporeal membrane oxygenation (ECMO)		84 (44.0)
Pumpless extracorporeal membrane oxygenation (pECLA)		20 (10.5)
Renal replacement therapy (24 h available)		177 (92.7)
Advanced hemodynamic monitoring (PiCCO, Swan-Ganz-catheter)		180 (94.2)
Advanced respiratory monitoring (NAVA, EIT, etc.)		57 (29.8)
Adaptive ventilation modes (NAVA, PAV, PAV+, etc.)		83 (43.5)
NO inhalation therapy		70 (36.6)
Cytokine elimination procedures		78 (40.8)
Describe the discontinuation criteria for NIV ventilation in COVID-19 patients.	165	
Consciousness disorder		145 (87.9)
Respiratory rate		135 (81.8)
Clinical assessment of the respiratory work		141 (85.5)
Rapid-Shallow-Breathing-Index		46 (27.9)
CO_2_ elimination disorder		128 (77.6)
Horovitz/oxygenation index		136 (82.4)
Measurement—work of breathing		23 (13.9)
If you are using RSBI as a discontinuation criterion for NIV therapy, explain your threshold. (mmHg)	31	105.97 ± 31.05
If you are using work of breathing as a discontinuation criterion for NIV therapy, explain your threshold. (J/L)	2	16.00 ±19.80
What alternative procedures are used instead of invasive ventilation in your ICU for critically ill COVID-19 patients. (*)	165	
Oxygen therapy only		32 (19.4)
High-Flow-nasal Oxygen (HFNC)		149 (90.3)
Conventional non-invasive ventilation via mask		147 (89.1)
Alternative NIV-Interface (helmets etc.)		45 (27.3)
If you use an HFNC, what flow rates are used in critically ill COVID-19 patients?	165	
HFNC as usual		128 (77.6)
Reduced flow rates compared to non-COVID to reduce aerosol production		24 (14.5)
No HFNC due to potential aerosol exposure for personnel		11 (6.7)
In COVID-19 patients * with severe ARDS, are you already early aiming for spontaneous breathing?	141	
Yes		89 (63.1)
No		23 (16.3)
Only in individual cases		28 (19.9)

## Data Availability

The dataset used and analyzed during the current study is available from the corresponding author on reasonable request.

## Abbreviations

COVID-19	Coronavirus disease-19
CEOSys	COVID-19 Evidence ecosystem
DIVI	German Interdisciplinary Association for Intensive Care and Emergency Medicine
ECMO	extracorporeal membrane oxygenation
PEEP	positive end-expiratory pressure
NUM	Nationales Forschungsnetzwerk der Universitätsmedizin
BMBF	Federal Ministry of Education and Research of Germany
SARS-CoV-2	severe acute respiratory syndrome coronavirus 2
ARDS	acute respiratory distress syndrome
ICU	Intensive Care Unit
HFNC	high flow nasal cannula
MV	mean value
SD	standard deviation
pECLA	pumpless extracorporeal lung assist
NIPPV	non-invasive positive pressure ventilation
HCWs	health care workers
NMBAs	neuromuscular blocking agents

## Appendix A

E-Mail to the leading physicians of German ICUs. Original translated from German:
Dear Colleagues,
to meet the challenge of the current Corona pandemic, 21 universities and 4 non-university partners have joined together to form an interdisciplinary consortium within the CEOsys collaborative project (COVID-19 Evidence Ecosystem to Improve Knowledge Management and Translation).
The aim of this consortium is to review scientific findings on COVID-19 pandemic management as quickly as possible in a quality-assured and independent manner for relevance and to make them available as updated overviews of results (“living evidence syntheses”).
The task of the intensive care physicians involved in this project (signatories) is to align these evidence syntheses with the pressing issues of their daily work and then to prepare and present them oriented to the specific preferences regarding information channel and format of the different target groups (nursing and medical intensive care staff) to achieve the broadest possible implementation of the generated knowledge.
For us to jointly achieve the goal of providing the best possible care for COVID-19 patients *, we ask that you.
1. As the medical director of your ICU, to complete the survey once per ICU (you can indicate your management function in the initial question), so that we can prioritize the topics of the evidence syntheses based on the structural data on current treatment standards in German ICUs in a way that is as demand-oriented as possible. During the survey, you have the option to voluntarily provide contact information. By participating in this structural data collection, we have the possibility to name you as a cooperation partner in a corresponding publication and to contact you regarding future participation in follow-up projects.
2. To forward the survey link as widely as possible to your intensive care staff (medical, nursing and other assisting staff) in your own hospital, so that we can take group-specific barriers and needs into account when imparting knowledge on the subject of intensive care medicine. In this survey, there is no possibility on our part to link the answers given here with those of the medical management!
3. Finally, we would like to ask you to forward this mail including the survey link to non-university hospitals in your catchment area to generate as comprehensive a data pool as possible.
The survey will take approximately 6-8 min to complete.
It is an anonymous data collection, conclusions on clinic or person or the linking of submitted answers (e.g., medical management with intensive care staff) are not possible!
The Intensive Care COVID-19 Treatment: Standards of Care and Individual Preferences in Knowledge Transfer survey is launched with the following link: https://www.soscisurvey.de/covid-evidenz1/?q=ITS (survey now offline)
(Survey period up to and including 31 December 2020).
We sincerely thank you in advance for your valued support!
With collegial regards,
for the working groups AP6, AP7 and TF3 of the CEOsys:
the CEOsys coordination in Freiburg (Prof. Dr. Jörg Meerpohl)
the Clinics of Anesthesiology and Intensive Care Medicine of the University Hospitals of
Würzburg (Prof. Dr. med. Patrick Meybohm, Prof. Dr. med. Peter Kranke, Maria Popp),
Leipzig (Priv.-Doz. Dr. med. habil. Sven Laudi, Falk Fichtner, MD, and Christian Seeber, MD),
Göttingen (Prof. Dr. med. Onnen Mörer, Dr. med. Steffen Dickel and Clemens Grimm),
And the German Interdisciplinary Association for Intensive Care and Emergency Medicine (DIVI).
For more information, please visit:
https://www.netzwerk-universitaetsmedizin.de/projekte/ceo-sys (accessed on 28 July 2021).
http://covid-evidenz.de/ (accessed on 28 July 2021)
Results of the online survey. Data in absolute number or mean value ± standard deviation. The original questions were asked in German. English translations are shown here. (*) for questions with multiple answers possible.
